# First person – Elise McKean

**DOI:** 10.1242/bio.062511

**Published:** 2026-02-17

## Abstract

First Person is a series of interviews with the first authors of a selection of papers published in Biology Open, helping researchers promote themselves alongside their papers. Elise McKean is first author on ‘ Drug repurposing to combat multidrug-resistant hookworm’, published in BiO. Elise is a PhD candidate in the lab of Dr Damien O'Halloran at The George Washington University, USA, investigating the molecular mechanisms that impart drug resistance in hookworm.


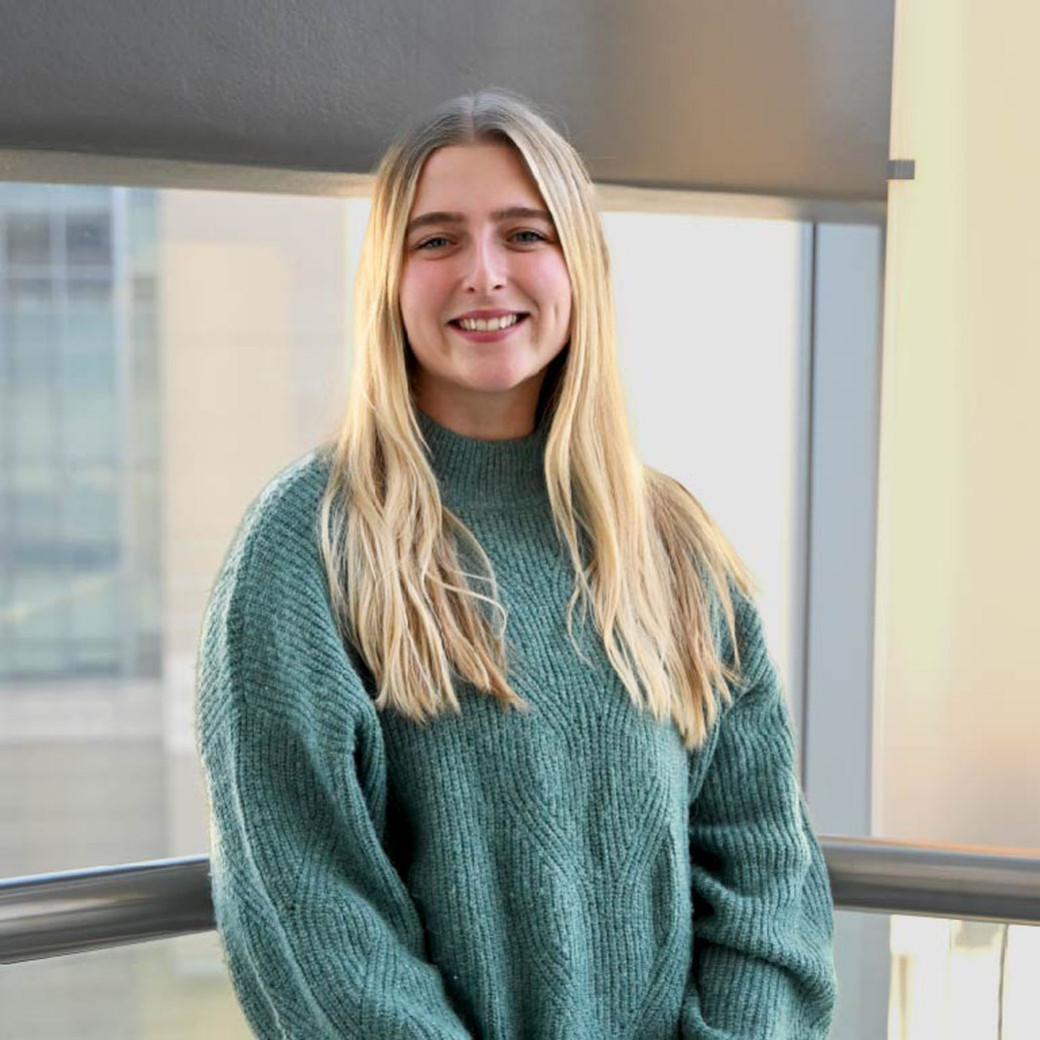


**Elise McKean**

**Describe your scientific journey and your current research focus**

I earned my bachelor's degree in ecology, evolution, and natural resources from Rutgers University in 2019, where I had the opportunity to work in several different research environments. I completed an internship with the New Jersey Division of Fish & Wildlife conducting bobcat population surveys, worked at an aquaculture research institute in Cape May selecting for heat- and salinity-tolerant shellfish, and assisted a PhD student in a parasite ecology laboratory. From my current research interests, it is probably clear which of these experiences had the greatest impact: the complexity of interactions between parasites and their hosts was difficult to beat.

After graduating, I took a position as a lab manager at The George Washington University in Dr John Hawdon's lab, where I met my current principal investigator, Dr Damien O'Halloran. Their laboratories work closely together, using the model organism *Caenorhabditis elegans* and the dog hookworm *Ancylostoma caninum* to investigate the causes of drug resistance and the differences between free-living and parasitic nematodes. My research now focuses primarily on the mechanisms of drug resistance in *A. caninum* and on identifying potential treatment options for isolates that exhibit resistance to all currently available classes of anthelmintic drugs.

**Who or what inspired you to become a scientist?**

Several people have inspired me over the course of my life, some whom I admired from afar and others whom I have known personally. Growing up, I watched Planet Earth and Blue Planet with my family, and David Attenborough's narration of the natural world completely captivated me. Seeing how diverse life on this planet is ultimately sealed my interest in the natural sciences. During my undergraduate program, I was required to complete a certain number of lab-based credit hours, and while searching for a position to fulfil that requirement, I came across a role in a parasite ecology laboratory. The PhD candidate I worked for, Rita Grunberg, became my first close mentor who was pursuing a doctorate. She was the first person to tell me that this was something I could do, and she helped me build both my confidence and my ability to think like a scientist. Watching her successfully navigate the latter half of her PhD, defend her thesis, and go on to work as a postdoctoral researcher made a future in academia feel far more accessible to me. I credit much of where I am today to her guidance and encouragement.

we discovered that flutamide, a drug currently prescribed for other medical conditions, can also kill parasitic worms

**How would you explain the main finding of your paper?**

Developing brand-new medicines takes a long time and costs a great deal of money, in part because every new drug must go through extensive testing to make sure it is safe for people to use. To help work around this problem, we looked for new uses for medicines that are already approved and known to be safe. Using computer-based tools to analyse large amounts of data, we discovered that flutamide, a drug currently prescribed for other medical conditions, can also kill parasitic worms.

**What are the potential implications of this finding for your field of research?**

This finding shows that computer-based approaches can be used to identify compounds that are effective against hookworms, even in populations that no longer respond to standard treatments. Beyond the immediate potential for drug discovery, these results may also help us better understand hookworm biology. By studying how these compounds work inside the worms, we can gain new insight into the biological pathways that are important for their survival.

**Figure:**
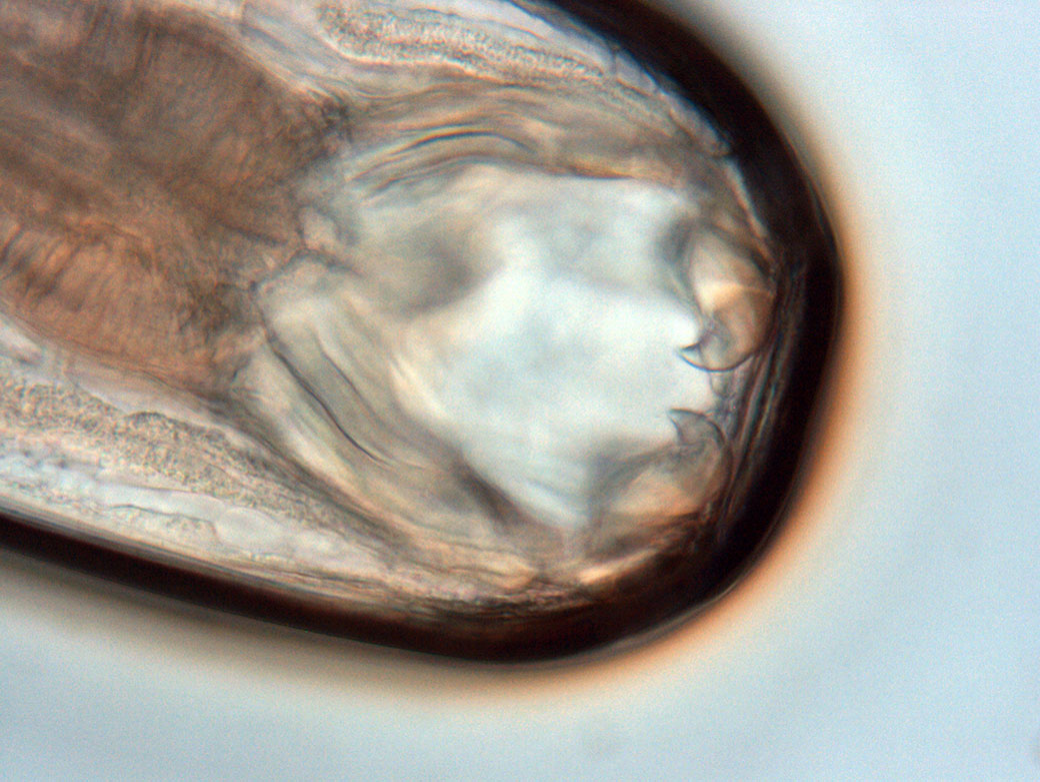
A photo of an adult *A. ceylanicum* hookworm baring its teeth, note the larger set of outer ventral teeth which is a defining morphological characteristic of this species.

**Which part of this research project was the most rewarding?**

The most rewarding part of this project came when we counted the screening egg hatch assays. This was the real test to see if the machine learning predictions were working as we had hoped. Seeing that a compound identified in our very first round of screening could block eggs from hatching was incredibly exciting and made the entire effort feel worthwhile.

**What do you enjoy most about being an early-career researcher?**

My favourite thing about being an early-career researcher is that my primary job right now is to try. Whenever I come across something that I think is interesting, I have the freedom to explore it and see where it leads. I love being able to message my advisor to ask if he has time for me to run an idea by him or going back and forth with my lab mate when one of us thinks of something new. Having the space to pursue ideas as they arise is what makes being in graduate school such a unique and rewarding experience.

interacting with as many researchers as possible is one of the best things you can do for yourself, especially early in your career

**What piece of advice would you give to the next generation of researchers?**

Talk to people. I think that many of my peers feel intimidated about reaching out to other researchers, whether within their own department or at other universities in the same field. But interacting with as many researchers as possible is one of the best things you can do for yourself, especially early in your career. One of the most important resources a research institution provides is an environment where you are surrounded by curious individuals who also happen to be experts in their subject matter.

**What's next for you?**

More data collection, and more time spent trying to figure out these worms, realistically, with them often staying one step ahead of me. I am planning to start writing my thesis toward the end of this year. For now, my goal is to stay in academia, and I plan to begin looking for postdoctoral positions once I have a date set for my defence. Ideally, I would like to work in a lab with a strong balance between computational and wet-lab research.
